# Heat Inactivation of Different Types of SARS-CoV-2 Samples: What Protocols for Biosafety, Molecular Detection and Serological Diagnostics?

**DOI:** 10.3390/v12070735

**Published:** 2020-07-07

**Authors:** Boris Pastorino, Franck Touret, Magali Gilles, Xavier de Lamballerie, Remi N. Charrel

**Affiliations:** Unité des Virus Émergents (UVE: Aix-Marseille Univ-IRD 190-Inserm 1207-IHU Méditerranée Infection), Aix-Marseille University, 13005 Marseille, France; boris.pastorino@univ-amu.fr (B.P.); Franck.touret@univ-amu.fr (F.T.); magali.gilles@univ-amu.fr (M.G.); xavier.de-lamballerie@univ-amu.fr (X.d.L.)

**Keywords:** SARS-CoV-2, coronavirus, heat inactivation, COVID-19, serology, ELISA, neutralization, virus neutralization test

## Abstract

Standard precautions to minimize the risk of SARS-CoV-2 transmission implies that infected cell cultures and clinical specimens may undergo some sort of inactivation to reduce or abolish infectivity. We evaluated three heat inactivation protocols (56 °C-30 min, 60 °C-60 min and 92 °C-15 min) on SARS-CoV-2 using (i) infected cell culture supernatant, (ii) virus-spiked human sera (iii) and nasopharyngeal samples according to the recommendations of the European norm NF EN 14476-A2. Regardless of the protocol and the type of samples, a 4 Log_10_ TCID50 reduction was observed. However, samples containing viral loads > 6 Log_10_ TCID_50_ were still infectious after 56 °C-30 min and 60 °C-60 min, although infectivity was < 10 TCID_50_. The protocols 56 °C-30 min and 60 °C-60 min had little influence on the RNA copies detection, whereas 92 °C-15 min drastically reduced the limit of detection, which suggests that this protocol should be avoided for inactivation ahead of molecular diagnostics. Lastly, 56 °C-30 min treatment of serum specimens had a negligible influence on the results of IgG detection using a commercial ELISA test, whereas a drastic decrease in neutralizing titers was observed.

## 1. Introduction

Since December 2019, measures to reduce person-to-person transmission of SARS-CoV-2 have been implemented in an attempt to control the COVID-19 outbreak. Tremendous efforts are being done by an increasing number of laboratory personnel working daily with infectious samples, and thus are heavily exposed to the risk of infection [1,2,3]. Accordingly, the WHO introduced laboratory guidelines to mitigate this risk for diagnosis and research activities [4]. Nonetheless, laboratory workers processing clinical samples will continue to be exposed to infectious SARS-CoV-2 [5]. Moreover, even if this enveloped virus is supposed to be fragile, it can persist in the environment for extended periods of time [6], and understanding the potential effect of heat inactivation of the pandemic SARS-CoV-2 coronavirus is also relevant to elaborate proper public intervention measures. In this work, we evaluated three heat inactivation protocols on different types of SARS-CoV-2 infectious samples in order to determine the efficacy in reducing the infectivity and the influence of these protocols on both the detection of viral RNA and the results of two serological assays.

## 2. Materials and Methods

### 2.1. Virus Strain and Titration

Experiments were performed in BSL3 facilities using a clinical SARS-CoV-2 strain (Ref-SKU: 026V-03883) isolated at Charite University (Berlin, Germany) and obtained from the European Virus Archive catalog (EVA-GLOBAL H2020 project) (https://www.european-virus-archive.com). The strain was inoculated at a 0.001 MOI in 90% confluent Vero-E6 cells (ATCC number CRL-1586) and incubated at 37 °C for 24–48 h, after which the medium was changed and incubation continued for 24 h; then, the supernatant was collected, clarified by spinning at 1500× *g* for 10 min, supplemented with 25mM HEPES (Sigma-Aldrich, Lyon, France), and aliquots were stored at −80 °C. One aliquot was thawed and used for titration using 50% tissue culture infectivity dose (TCID_50_); briefly, when cells were at 90% confluence, six replicates were infected with 150μL of ten-fold serial dilutions of the virus sample, and incubated for 4 days at 37 °C under 5% CO_2_. Cytopathic effect (CPE) was read using an inverted microscope, and infectivity was expressed as TCID_50_/mL based on the Karber formula [7]. All samples were quantified by end-point titration on Vero E6 cells with a limit of detection of about 10^0.5^ TCID50/mL (3.16 TCID50/mL).

### 2.2. Samples Used for Heat Inactivation

Three types of sample were used for assessing the efficacy of heat inactivation protocols: (i) SARS-CoV-2 infected Vero-E6 cell supernatants (with or without supplementation with 3g/L bovine serum albumine [BSA]), (ii) nasopharyngeal samples (NPS) collected in patients, (iii) and sera from blood donors (BD); the two latter were collected before the COVID-19 pandemic period, and were negative for SARS-CoV-2 RNA and for SARS-CoV-2 antibodies, respectively.

NPS were collected into 1 mL of viral transport media (Virocult^®^, Sigma). They were pooled in order to constitute a homogeneous material that was spiked with infectious SARS-CoV-2 to a final titer ranging from 10^5^ to 10^6^ TCID_50_/mL depending on the sample type. Spiked material was then distributed in 300 µL aliquots before performing the different heating protocols. The same approach was applied to BD sera.

### 2.3. Heat Inactivation of SARS-CoV-2 Samples

The virucidal activity of different heat protocols was determined according to the European Standards NF EN 14476-A2 (https://www.analytice.com/en/nf-en-14476-laboratory-biocide-efficacy-test/). Briefly, a 300-µL sample containing 10^5^ to 10^6^ TCID_50_/mL was incubated in a pre-warmed dry heat block using either of the three following protocols: 56 °C-30 min, 60 °C-60 min and 92 °C-15 min, after which the treated sample was immediately titrated (TCID_50_) and tested for RNA copies (Table 1). Virus titration and RT-qPCR were performed before and after heating to measure the viral load reduction factor and variation in RNA copies. Samples were tested in duplicates (cell supernatants) or in six replicates (NPS and BD sera). For NPS and BD sera, the 92 °C-15 min protocol was not performed because of its poor suitability for practical applications in clinical microbiology laboratories [8].

### 2.4. Integrity of SARS-CoV-2 RNA before and after Heat Inactivation

Heat inactivated samples and control samples were extracted using the Qiacube HT and the Cador pathogen extraction kit (both from Qiagen, Venlo, The Netherlands). Viral RNA was quantified by RT-qPCR (qRT-PCR EXPRESS One-Step Superscript™, ThermoFisher Scientific, Waltham, Massachusetts) (10 min-50 °C, 2 min-95 °C, and 40 times 95 °C-3 s/60 °C-30 s) using serial dilutions of a T7-generated synthetic RNA standard. Primers and probe target the *N* gene (Fw: GGCCGCAAATTGCACAAT; Rev: CCAATGCGCGACATTCC; Probe: FAM-CCCCCAGCGCTTCAGCGTTCT-BHQ1. The calculated limit of detection was 10 RNA copies per reaction.

### 2.5. Impact of 56 °C-30 min Heating on Results of Serological Assays

To address whether heating sera at 56 °C for 30 min may affect the results observed with two serological assays, a total of 38 SARS-CoV-2 positive human sera were selected, processed and reanalyzed comparatively as detailed hereunder.

#### 2.5.1. Detection of SARS-CoV-2 IgG by ELISA

The semi-quantitative anti-SARS-CoV-2 ELISA for immunoglobulin class G (EI 2606-9601 G, Euroimmun AG, Lübeck, Germany) was used as recommended by the manufacturer. The optical density (OD) was detected at 450 nm, and a ratio of the reading of each sample to the reading of the calibrator was calculated for each sample (OD ratio). Samples were considered positive when OD ratio >1.1.

#### 2.5.2. Detection of SARS-CoV-2 Neutralizing Antibodies

A virus neutralization test (VNT) was performed as previously described [9]. Briefly, VNT was performed in a 96-well format, using Vero-E6 cells and virus strain described in 2.1. Two-fold serial dilutions of sera were mixed with 100 TCID_50_, resulting in final serum dilutions ranging from 1/20 to 1/160, and incubated for 1 h at 37 °C. Serum+virus was transferred onto the confluent cell monolayer, and incubated at 37 °C in a 5% CO_2_ atmosphere. Positive and negative control wells, containing virus+cells and cells only, respectively, were included in each series. After 4 days, the plates were examined for the presence (no neutralization) or absence (neutralization) of CPE using an inverted microscope.

## 3. Results

### 3.1. Heat Inactivation of SARS-CoV-2 Samples

#### 3.1.1. Heat Inactivation of SARS-CoV-2 Infected Cell Supernatant

The three protocols resulted in a clear decrease in the infectivity after treatment with a reduction factor that was equal or higher than 5 Log_10._ Accordingly, based on the NF EN 14476-A2, they all were considered as virucidal (Table 1). However, some of the samples treated with the 56 °C-30 min and 60 °C-60 min protocols remained infectious, whereas virus recovery was not possible with the samples treated with the 92 °C-15 min protocol. There was no difference between clean or dirty conditions, meaning that an interfering substance (i.e., high level of proteins) did not hamper the efficacy of heating as an inactivation method. Interestingly, the 92 °C-15 min protocol resulted in a drastic reduction in the detectable RNA copies (ΔCt > 5) before and after heat treatment (8 × 10^6^ vs. 1.6 × 10^5^ RNA copies). This is an important point because it should alert us to the fact that heat-inactivated samples may not be suitable for viral RNA detection due to the possible decrease in the sensitivity and increase in the limit of detection (LoD), with expected false negative results.

#### 3.1.2. Heat Inactivation of SARS-CoV-2 Spiked Nasopharyngeal Samples

The two protocols (56 °C-30 min and 60 °C-60 min) resulted in a clear drop in infectivity (>5 Log_10_ reduction) (Table 1). Both protocols did not affect the number of detectable RNA copies samples (Ct Var < 2).

#### 3.1.3. Heat Inactivation of SARS-CoV-2 Spiked Blood Donor Sera

The two protocols (56 °C-30 min and 60 °C-60 min) resulted in a clear drop in infectivity (>5 Log_10_ reduction) (Table 1). Both protocols did not affect the number of detectable RNA copy samples, although 56 °C-30 min had a lower impact on the LoD (Ct Var Ct < 1 vs. < 2).

### 3.2. Impact of 56 °C-30 min Protocol on the Results of Serological Assays

A total of 38 sera from patients with RT-qPCR-established SARS-CoV-2 infection were collected during the convalescent phase and tested using both serological assay in order to compare the results observed with and without heating at 56 °C-30 min. The aim was to address whether 56 °C-30 min could influence the results of two serological assays or not.

#### 3.2.1. ELISA

**Figure 1:**
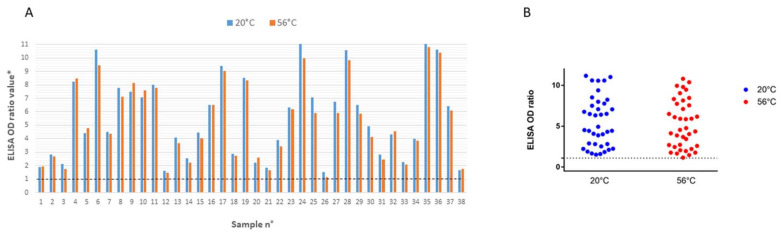
Impact of heat treatment at 56 °C-30 min on the SARS-CoV-2 ELISA EuroImmun IgG assay compared with the same sera maintained at ambient temperature (20 °C). Panel (**A**), histogram representation. (**B**): point cloud representation. Positivity threshold is represented by the discontinuous line.

There was no difference in the results observed with the two protocols, neither in the final results (negative/positive with 1.1 cut-off threshold) nor when OD ratios were compared (Figure 1). Together, these results demonstrate that 56 °C-30 min had a negligible influence on ELISA results. In addition, ten sera collected from patients during the pre-pandemic period in 2019 were negative with and without 56 °C-30 min treatment [10].

#### 3.2.2. Virus Neutralization Test

**Figure 2:**
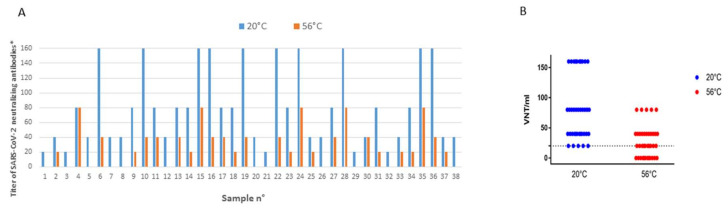
Impact of heat treatment at 56 °C-30 min on the SARS-CoV-2 neutralization assay compared with the same sera maintained at ambient temperature (20 °C). Panel (**A**), histogram representation. (**B**): point cloud representation. * Serum specimens with a titer ≥ 20 were considered positive.

There was a clear difference when using heated sera compared to unheated sera (Figure 2). Lower titers were observed after the 56 °C-30 min protocol in 36 of the 38 tested sera; only sera #4 and #30 showed equal titers with and without heating. Sixteen sera showed titer reduction of one dilution, whereas the remaining 20 sera showed titers that were decreased by at least 2 dilutions after heating. Considering a qualitative analysis (positive/negative using 20 as the cut-off titer), 12 sera that were positive in the absence of heat treatment shifted into the negative group, leading to a total of 31% false negatives in our series.

## 4. Discussion

Understanding the potential effect of heat inactivation on the novel coronavirus SARS-CoV-2 is important in environmental and laboratory conditions in order to elaborate adapted biosafety protocols. Owing to the contagiousness of SARS-CoV-2, heat inactivation can be considered (i) for abolishing virus infectivity or (ii) for reducing the infectivity by viral load reduction. In this study, we used three different types of sample in order to mimic situations encountered in laboratory and non-laboratory environments. SARS-CoV-2-infected cell culture supernatant is commonly manipulated in BSL3 laboratories where the virus is grown for research or diagnostic activities. Nasopharyngeal samples (NPS) are clinical specimens that are manipulated by medical and laboratory personnel in routine care and diagnostic activities. Lastly, serum specimens are commonly manipulated in various types of laboratory activities in and outside of the field of virology. Therefore, addressing the effects of three heating protocols onto these different types of samples is important to better understand their possible use to reduce or suppress the infectivity of the SARS-CoV-2 and to define biosafety measures. The possible influence of heating protocols on the results of virological techniques is also important to address to avoid a loss of sensitivity and false negative results in diagnostic procedures. As 60 °C-60 min can be detrimental for serology and 92 °C 15 min results in a clear drop in RNA quantity detection, these protocols were excluded from certain modules of our study.

According to the European norm NF EN 14476-A2, the protocols tested in this study can be considered as virucidal, i.e., achieving a 4 Log_10_ reduction in infectivity for all tested samples (cell supernatant, virus-spiked serum or nasopharyngeal samples). However, in our experimental conditions, samples containing viral loads > 6 Log_10_ TCID_50_ remain infectious after 56 °C-30 min and 60 °C-60 min, although the risk of infection at the individual level with samples containing viral loads below 10 TCID_50_ is not established. In any case, the postulate is that if a given viral load can infect the cell monolayer, the risk of human infection exists. Interestingly, the efficacy of the 56 °C-30 min and 60 °C-60 min protocols are in line with results observed using canine coronavirus and mouse hepatitis coronavirus (3.88 to 4.51 Log_10_ reduction factor) with 60 °C for 30 min [11]. However, 56 °C-30 min appears much less efficient on SARS-CoV-2 than on transmissible swine gastroenteritis virus, an alpha coronavirus, showing a reduction in infectivity of >7.5 Log_10_ units [12]. This suggests that the inactivation of clinical samples ahead of molecular diagnosis should also consider chemical inactivation as an alternative to heat inactivation for SARS-CoV-2 diagnostics [13].

In this study, the lack of impact of 56 °C-30 min and 60 °C-60 min protocols on RNA copies detection confirms recently reported results [14]; however, in contrast, the latter reported a lower decrease in copy number detectable after 95 °C-3 min (ΔCt = 2.2) compared to the ΔCt > 5 after 92 °C-15 min we observed. This is not totally unexpected because of differences in the two protocols. This also raises questions about the possibility to inactivate high viral loads within respiratory samples by short heating at high temperature, an option that is increasingly debated with protocol replacing nucleic acid extraction by heat denaturation [15,16,17]. It is clear that additional studies are needed to elaborate heating protocols achieving infectivity loss together with unchanged detectable copy number.

Complement inactivation through 56 °C-30 min heating is common before ELISA serology. Additionally, 60 °C-60 min and 56 °C-30 min are used for reducing the potential infectivity of the samples processed for serology [18]; however, either of these treatments may have a deleterious impact on the results qualitatively or quantitatively [19]. The aim was to address whether 56 °C-30 min could influence the results of two serological assays or not. In contrast with Hu et al. [19], our results suggest that 56 °C-30 min does not affect, either qualitatively nor quantitatively, the detection of SARS-CoV-2 specific IgG using a commercially available ELISA test. However, 56 °C-30 min had a noticeable effect on the detection of neutralizing antibodies. To the best of our knowledge, this has not been described previously, either for coronaviruses or for other viruses. One recent study reported that combining 56 °C-30 min with phosphate buffered saline-Tween20 (0.3%) supplementation (vol/vol) with (0.15% final concentration) influenced neutralization titers, specifically when they ranged from 20 to 80 (up to 30% of sera showed measurable impact on the result) [20]. Although we have no evidence-based information to explain this phenomenon, it could be due to (i) the partial heat denaturation of antibodies, changing their ability to neutralize the virus but not the antigen binding or (ii) the presence in the serum of components, other than antibodies, that are heat-sensitive, such as the complement as described for Junín virus and human cytomegalovirus [21,22]. Further studies are needed to assess whether this phenomenon is virus-dependent or more widely observed.

In conclusion, the results observed in this study should be taken into consideration for sorting serum samples according to the subsequent serological assays to be performed. Upon reception, aliquoting of serum samples is necessary to allow different processes downstream. Since ELISA is commonly used as a screening test before ELISA-positive sera are confirmed by neutralization assay, performing VNT using heat-inactivated sera can result in reduced titers and in false negative results. Therefore, we advocate that heat inactivation is performed on aliquots before ELISA and that confirmation VNT is performed with unheated aliquots.

Finally, this study should help to choose the best-suited protocol for inactivation in order to prevent the exposure of laboratory personnel in charge of direct and indirect detection of SARS-CoV-2 for diagnostic or research purposes.

## Figures and Tables

**Table 1:** Heat inactivation of three types of samples and impact on the RNA detection.

Type of Sample	Heating Protocol	Viral Titer (TCID_50_/mL) ^a^			Log_10_ Reduction Factor (LRF)	Number of RNA Copies		Ct Var ^b^
		Before Heat Inactivation	After Heat Inactivation			Before Heat Inactivation	After Heat Inactivation	
			No BSA	3g/L BSA				
**SARS-CoV-2 infected cell supernatant ^c^**	56 °C, 30 min	3.3 ± 2.3 × 10^6^	8.5 ± 7	ND (0/2) ^e^	5 < LRF < 6	8.01 × 10^6^	5.16 × 10^6^	<0.7
	60 °C, 60 min	3.3 ± 2.3 × 10^6^	ND (0/2)	5 ± 2.8	5 < LRF < 6	8.01 × 10^6^	4.54 × 10^6^	<0.8
	92 °C, 15 min	3.3 ± 2.3 × 10^6^	ND (0/2)	ND (0/2)	LRF > 6	8.01 × 10^6^	1.6 × 10^5^	>5
**SARS-CoV-2 spiked nasopharyngeal sample** **^d^**	56 °C, 30 min	3.5 ± 2.3 × 10^5^	ND (0/6)		LRF > 5	7.5 × 10^5^	2.1 × 10^5^	<1.5
	60 °C, 60 min	3.5 ± 2.3 × 10^5^	ND (0/6)		LRF > 5	7.5 × 10^5^	1.5 × 10^5^	<2
**SARS-CoV-2 spiked blood donor sera ^d^**	56 °C, 30 min	3.5 ± 2.3 × 10^5^	ND (0/6)		LRF > 5	7.5 × 10^5^	3.5 × 10^5^	<1
	60 °C, 60 min	3.5 ± 2.3 × 10^5^	ND (0/6)		LRF > 5	7.5 × 10^5^	1.5 × 10^5^	<2

^a^ Mean value ± SD according to the Karber formula as described in the Materials and Methods section; ^b^ Ct Var, Cycle threshold variation = Ct value (before inactivation)-Ct value (after inactivation); ^c^ two replicates; ^d^ six replicates; ^e^ not detected (both replicates or all 6 replicates depending upon the type of sample). All samples were quantified by end-point titration on Vero E6 cells with a limit of detection of about 10^0.5^ TCID_50_/mL.
